# Modification of second cancer risk after malignant melanoma by parental history of cancer

**DOI:** 10.1038/sj.bjc.6604489

**Published:** 2008-07-15

**Authors:** H Zhang, J Lorenzo Bermejo, J Sundquist, K Hemminki

**Affiliations:** 1 Division of Molecular Genetic Epidemiology, German Cancer Research Center (DKFZ), Heidelberg, Germany; 2 Center for Family and Community Medicine, Karolinska Institute, Alfred Nobels alle 12, 14183 Huddinge, Sweden

**Keywords:** familial risk, second cancer, genetic susceptibility, melanoma

## Abstract

The Swedish Family-Cancer Database was used to quantify the incidence of second tumours in melanoma patients with a parental history of cancer. Patients with parents affected by melanoma showed a 32.3-fold risk of second primary melanomas, which was greater than a multiplicative interaction.

Compared with other skin cancers, cutaneous melanoma (CM) is a rare disease, with an incidence rate of 14 cases per 100 000 in Sweden, one of the highest rates in Europe (de Vries and Coebergh, 2004). In contrast, CM is responsible for 80% of all skin cancer-related deaths (Miller and Mihm, 2006). The improvements in survival after melanoma have resulted in an increased incidence of subsequent primary tumour survival (Bhatia *et al*, 1999). Melanoma patients are prone to develop second tumours in the breast, prostate, kidney and skin, and particularly, second primary melanomas (Schmid-Wendtner *et al*, 2001; Goggins and Tsao, 2003), but those with a parental history of cancer have not been ascertained separately. The present population-based study investigates the risk of type-specific second tumours after diagnosis of melanoma and expands on the familial risk of subsequent cancers. Apart from its importance for clinical counselling and prevention, an understanding of the role of parental history in multiple cancers may help to understand cancer aetiology.

## Materials And Methods

The Swedish Family-Cancer Database, which was created in 1995 and last updated in 2006, includes a total of 11. 5 million individuals. The Database covers offspring born or immigrated after 1931 with information on biological parents (Hemminki *et al*, 2006). In this study, cancers were classified according to the 4-digit diagnostic code from the seventh version of the International Classification of Disease (ICD-7). Tumours are compulsorily reported in Sweden and the agreement between clinical and cytological or histological diagnoses is close to 100%.

In the present retrospective cohort study, follow-up started at diagnosis of melanoma in a member of the offspring generation and it ended at occurrence of death, emigration, the diagnosis of any subsequent malignancy or 31 December 2004 (close date of the study). Two types of parental history were investigated: a parental history of melanoma (one of the two parents of the individual diagnosed with malignant melanoma) and a concordant parental history (one of the parents affected by the same type of cancer as the second primary cancer in the offspring). Standardized incidence ratios (SIR) were used to measure the relative risk of second cancers and they were calculated as the ratio of observed (O) to expected (E) number of cases. The expected numbers were computed from the first primary cancer incidence rates taking into account age (5-year intervals), region (four regions), calendar period (1960–1964, 1965–1969,…, 2000–2004) and socio-economic status (six groups). A Poisson distribution of the number of cases was assumed. Multiplicative interaction indexes (MIIs) and interaction contrast ratios (ICRs) were used to investigate the possible interaction between ‘individual history of melanoma’ and ‘parental history of cancer’. If SIR_melanoma_ represents the relative risk of cancer after melanoma, SIR_ph_ the relative risk in individuals with a parental history of cancer and SIR_melanomaxph_ the relative risk in melanoma patients with a parental history, MII=SIR_melanomaxph_/(SIR_melanoma_xSIR_ph_) and ICR=SIR_melanomaxfh_-SIR_melanoma_-SIR_ph_+1. MII1 suggests departure from multiplicativity and ICR0 indicates departure from additivity. Confidence intervals and *P*-values for MII and ICR were calculated by bootstrapping using 10 000 replications. All statistical analyses were carried out in SAS 9.1.

## Results

We identified 15 581 individuals in the offspring generation diagnosed with malignant melanoma before the age of 73 years (registration of individuals started in 1931 and the last update of the Database includes cancers diagnosed before year 2004). Among the patients, 1156 developed a subsequent cancer. Table 1 shows the SIRs for sites where at least 10 cases were observed. Compared with the general Swedish population, an increased risk of second cancer was observed for the breast (1.35), prostate (1.22), skin (melanoma 9.03 and squamous cell 3.48) and nervous system (1.66). Lymphohematopoietic neoplasms were also in excess, mostly because of an increased risk of non-Hodgkin's lymphoma with an SIR of 1.60. A parental history of melanoma increased the risk of second melanoma to an SIR of 32.3. The analysis of the interaction between individual and parental history of melanoma resulted in MII=1.46 (95% CI 1.06 to 2.03, *P*=0.03) and ICR=21.7 (95% CI 13.3–33.3, *P*<0.01), thus suggesting greater than multiplicative and greater than additive interaction effects.

Table 2 shows the relative risks of site-specific cancers for individuals with a parental family history of the particular cancer (first column) and the parental risks of the same (concordant) cancer after melanoma. For example, the relative risk of liver cancer was 1.3 for the offspring of liver cancer patients, but the SIR increased to 13.5 (two cases) when the offspring were melanoma patients. The relative risk of breast cancer in women with affected mothers was 1.64, and it increased to 2.51 among melanoma patients with a maternal history of breast cancer. The interaction between ‘individual history of melanoma’ and ‘maternal history of breast cancer’ did not significantly depart from multiplicativity or additivity (MII=1.13 (95% CI 0.79–1.64, *P*=0.29) and ICR=0.52 (95% CI −0.24–1.59, *P*=1.14)). Melanoma patients with a parental history of kidney cancer were at very high risk of developing second kidney cancers (SIR 13.1, three cases).

## Discussion

The present study, based on the nation-wide Swedish Family-Cancer Database, focuses on the risk of subsequent cancer after malignant melanoma in individuals with a parental history of cancer. In agreement with previous studies, melanoma patients were at an increased risk of second tumours, including melanomas and lymphomas. The reasons for these increases are probably manifold. Immune suppression induced by therapy, exposure to ultraviolet radiation and the primary disease could explain some cases, particularly for non-Hodgkin's lymphoma (Ebbesen, 1981; Romedahl *et al*, 1988; Hemminki *et al*, 2003). A novel result of this study was the increased risk observed for squamous cell carcinoma. This association has been previously observed in a reversed sequence: increased risk of melanoma after squamous cell carcinoma (Hemminki and Dong, 2000). In addition to treatment-related factors, unidentified susceptibility genes could explain these results to some extent. Inactivation of CDKN2A by genetic and epigenetic changes has been described in melanoma, squamous cell carcinoma, breast cancer and Hodgkin disease (Soufir *et al*, 1999; Brown *et al*, 2004; Debniak *et al*, 2007; Sinha *et al*, 2008). However, the association between germline mutations in CDKN2A and malignancies other than melanoma are still unclear. Mutations in MC1R, a low-penetrance melanoma-predisposing gene, could also be involved in squamous cell skin cancers and in Hodgkin's disease (Palmer *et al*, 2000; Box *et al*, 2001; Figl *et al*, 2007). The novel data from the present study refer to the risk of second concordant tumours in melanoma patients with a parental history of cancer. The risks were increased for second liver and kidney cancers after melanoma when the parents were diagnosed with liver or kidney cancer, respectively; however, the number of cases was small. More convincingly, a greater than additive and greater than multiplicative interaction was observed between parental and individual history of melanoma, with an expected SIR of 2.44 × 8.41=20.5 and an observed SIR of 32.3 (the SIR of second melanoma for individuals without a parental history was 8.41, 311 cases, 95% confidence interval: 7.50–9.40, data not shown). A study based on a previous version of the Database showed that, when mothers had breast cancer and fathers melanoma, the offspring was at a high risk of both breast cancer and melanoma (Hemminki and Vaittinen, 1997). The present data could mirror the effects of gene-environment interactions – for example, between CDKN2A mutations and UV-exposure – or the combinations among yet unidentified susceptibility genes.

## Figures and Tables

**Table 1:** Relative risks of second neoplasms after melanoma in the general population and in patients with a parental history of melanoma

	**All offspring**				**Offspring of melanoma patients**			
**Type of second cancer**	**N^a^**	**SIR^b^**	**(95% CI^c^)**		**N**	**SIR**	**(95% CI)**	
Any type	1156	**1.74**	1.64	1.85	53	**3.59**	2.69	4.69
*Solid cancers*	1089	**1.76**	1.66	1.87	51	**3.71**	2.76	4.88
Upper aerodigestive tract	16	1.50	0.86	2.45	1	4.12	0.00	23.6
Colorectum	54	0.99	0.74	1.29	1	0.88	0.00	5.06
Liver	11	1.19	0.59	2.14	0			
Pancreas	13	1.17	0.62	2.07	1	4.46	0.00	25.6
Lung	34	0.82	0.57	1.15	2	2.33	0.22	8.59
Breast	206	**1.35**	1.17	1.55	5	1.46	0.46	3.44
Endometrium	17	0.73	0.42	1.17	1	2.30	0.00	13.2
Ovary	16	0.83	0.47	1.34	0			
Prostate	111	**1.22**	1.01	1.47	3	1.51	0.29	4.48
Kidney	22	1.54	0.96	2.34	1	3.27	0.00	18.7
Urinary bladder	30	1.32	0.89	1.89	0			
Melanoma	343	**9.03**	8.10	10.0	32	**32.3**	22.1	45.6
Skin, squamous cell	53	**3.48**	2.61	4.56	1	2.99	0.00	17.1
Nervous system	43	**1.66**	1.20	2.24	2	3.14	0.30	11.6
								
*Lymphohaematopoietic neoplasms*	67	**1.50**	1.16	1.90	2	1.92	0.18	7.07
Non-Hodgkin's lymphoma	33	**1.60**	1.10	2.26	0			
Myeloma	11	1.66	0.83	2.98	0			
Leukaemia	16	1.06	0.60	1.72	1	2.85	0.00	16.3

a Number of melanoma patients with second neoplasms.

b Standardised Incidence Ratio.

c Confidence interval. Bold type represents statistical significance at the 0.05 confidence level.

**Table 2:** Parental relative risks of cancer and relative risks of second neoplasms in melanoma patients with a parental history of concordant (same site) cancer

	**Individuals with parents affected by concordant cancer**				**Melanoma patients with parents affected by concordant cancer**			
**Type of second cancer**	**N^a^**	**SIR^b^**	**(95% CI^c^)**		**N**	**SIR**	**(95% CI)**	
Any type	81548	**1.05**	1.05	1.06	629	**1.93**	1.78	2.09
*Solid cancers*	71104	**1.06**	1.05	1.07	557	**1.95**	1.79	2.12
Upper aerodigestive tract	49	1.67	1.26	2.22	0			
Colorectum	1573	**1.69**	1.61	1.78	3	0.76	0.14	2.25
Liver	60	**1.30**	1.01	1.69	2	**13.5**	1.28	49.8
Pancreas	85	**2.01**	1.62	2.50	1	4.82	0.00	27.7
Lung	647	**1.72**	1.58	1.86	1	0.56	0.00	3.20
Breast	3656	**1.64**	1.59	1.70	25	**2.51**	1.62	3.71
Endometrium	163	**2.16**	1.85	2.52	1	3.07	0.00	17.6
Ovary	176	**2.48**	2.13	2.88	0			
Prostate	2472	**1.88**	1.80	1.96	11	1.57	0.78	2.82
Kidney	119	**2.19**	1.82	2.62	3	**13.1**	2.47	38.7
Urinary bladder	279	**1.79**	1.58	2.01	2	3.25	0.31	12.0
Melanoma	438	**2.44**	2.22	2.68	32	**32.3**	22.1	45.6
Skin, squamous cell	172	**2.03**	1.74	2.36	2	4.04	0.38	14.8
Nervous system	257	**1.69**	1.50	1.92	1	2.23	0.00	12.8
								
*Lymphohaematopoietic neoplasms*	879	**1.38**	1.29	5	2.83	0.89		6.65
Non-Hodgkin's lymphoma	167	**1.86**	1.60	2.17	2	6.33	0.60	23.3
Myeloma	35	**2.43**	1.74	3.40	0			
Leukaemia	150	**1.87**	1.58	2.19	1	4.47	0.00	25.7

a Number of cases with a parental history.

b Standardised Incidence Ratio.

c Confidence interval. Bold type represents statistical significance at the 0.05 confidence level.
